# Dual trigger for final oocyte maturation in expected normal responders with a high immature oocyte rate: a randomized controlled trial

**DOI:** 10.3389/fmed.2023.1254982

**Published:** 2023-10-05

**Authors:** Meng-Han Yan, Zhen-Gao Sun, Jing-Yan Song

**Affiliations:** ^1^The First Clinical College, Shandong University of Traditional Chinese Medicine, Jinan, China; ^2^Reproductive Center of Integrated Medicine, The Affiliated Hospital of Shandong University of Traditional Chinese Medicine, Jinan, China

**Keywords:** normal ovarian responder, dual trigger, gonadotropin releasing hormone agonist, human chorionic gonadotropin, MII oocytes

## Abstract

**Objective:**

To evaluate whether dual trigger could improve reproductive outcomes in women with low oocyte maturation rates compare to human chorionic gonadotropin (hCG) trigger.

**Methods:**

This study included expected normal ovarian responders younger than 40 years old whose immature oocyte rate in the previous cycle was more than 50% at the reproductive center from July 2021 to November 2022. A total of 73 patients were enrolled at trigger, including 34 in the hCG trigger group and 39 in the dual trigger group (co-administration of gonadotrophin releasing hormone (GnRH) agonist and hCG, 40 and 34 h prior to oocyte retrieval, respectively). The primary outcome was oocyte maturation rate.

**Results:**

There was no significant difference in the number of oocytes retrieved between the two study groups, but the oocyte maturation rate was higher in dual trigger group (84.0% [14.0%] vs. 55.5% [19.8%], *p* < 0.001). Moreover, there were also higher cumulative pregnancy rate (69.4% vs. 40.0%, *p* = 0.035) and cumulative live birth rate (66.7% vs. 36.0%, *p* = 0.022) in dual trigger group.

**Conclusion:**

For normal responders with low oocyte maturation rates, the dual trigger may be more effective than the conventional hCG trigger.

**Clinical trial registration:**

ClinicalTrials.gov, identifier ChiCTR2100049292.

## Introduction

During the period preceding spontaneous ovulation, the oestradiol (E_2_) level secreted by the dominant follicle reaches its peak, triggering the surge of follicle stimulating hormone (FSH) and luteinizing hormone (LH) levels, both of which cooperate to promote oocyte maturation and excretion during the final stages of the process. It has been shown that hCG alone can be used in ovarian stimulation cycles using a GnRH antagonist (GnRH-ant) protocol for triggering maturation of the oocyte and induced meiosis/follicular maturation as a substitute for LH surge (1). As a result of the prolonged luteinization, hCG-only trigger is associated with a higher risk of ovarian hyperstimulation syndrome (OHSS) (2). Induction of oocyte maturation using gonadotrophin releasing hormone agonist (GnRH-a) has been shown to decrease OHSS incidence in comparison to hCG triggers. However, due to the lower total amount and shorter duration of endogenous gonadotropins (Gn) produced following GnRH-a stimulation, which led to the subsequent luteal insufficiency, there is a reduction in pregnancy rates and a higher rate of miscarriage (3, 4). This may be related to the possibility that triggering with GnRH-a or r-hCG results in distinct EV miRNA expression profiles and downstream biological effects in ovarian follicles (5). Addressing this issue with co-administration of GnRH-a and hCG, the dual trigger approach, both reduced the risk of OHSS in patients with a high ovarian response (6) and contributed to the comparable or even higher pregnancy rates compared with hCG trigger (7). In addition, dual triggers increased the number and quality of oocytes in normal responders, as well as the fertilization rate (8). Orvieto elucidated how to tailor each trigger mode to its appropriate subgroup of patients (9).

In addition, it is of interest that a new trigger regimen, co-administration of GnRH-a and hCG, 40 and 34 h prior to ovum pick-up (OPU), respectively, was used to prolong the time between trigger and OPU, with the success of obtaining mature oocytes, pregnancy, delivery in a patient with recurrent empty follicle syndrome (10). Furthermore, the studies relevant to normal responders who had a lower rate of mature oocytes (<50%) indicated that this dual trigger regimen resulted in a significant increase in the number of mature oocytes and transferable embryos obtained, as well as the proportion of oocytes obtained to the number of preovulatory follicles, but the difference in pregnancy rates was not conclusive (11, 12). Nevertheless, poor oocyte maturation rates are associated with lower clinical pregnancy and live birth rates according to recent studies (13).

Based on this, the present study was designed to investigate whether dual trigger could improve the MII oocytes rate and pregnancy outcomes in normal responders with poor oocyte maturation rates.

## Materials and methods

### Ethical approval of the study protocol

The reproductive ethics committee of the Affiliated Hospital of Shandong University of Traditional Chinese Medicine (TCM) certified this study as ethical (Identifier: SDUTCM/2021.7.26). All patients provided written informed consent. All treatments were undertaken in strict accordance with the Declaration of Helsinki 1964 and its later amendments.

### Study design

This study was a registered randomized controlled trial (RCT, http://www.chictr.org.cn/, identifier: ChiCTR2100049292) carried out in the reproductive center, affiliated hospital of Shandong University of Traditional Chinese Medicine, between July 2021 to November 2022 (14).

### Inclusion criteria

(1) Patients with an expected normal ovarian response (NOR) who had no previous history of cancellation of an *in vitro* fertilization / intracytoplasmic sperm injection (IVF/ICSI) cycle; (2) Patients with an expected NOR who had no previous history of poor ovarian response of an IVF/ICSI cycle; 6 ≤ AFC ≤ 15; 1.2 ng/mL ≤ AMH ≤ 3.5 ng/mL; Basal FSH < 10 mIU / ml; (3) Patients with <50% mature oocytes in the only one previous fresh IVF/ICSI cycle triggered with hCG; and (4) In the previous IVF/ICSI cycle, standard ovarian stimulation protocol was performed using a GnRH-ant protocol.

### Exclusion criteria

(1) Age ≥ 40 years old; (2) Patients with a body mass index (BMI) ≥ 30 kg/m^2^; (3) Individuals with high risk of OHSS during controlled ovarian stimulation; (4) Patients with endocrine or metabolic disorders; (5) Patients with untreated severe endometriosis, submucosal myoma, multiple endometrial polyps, pelvic inflammation, uterine malformation, Asherman syndrome and hydrosalpinx prior to embryo transfer (ET); and (6) Patients with abnormal immune function and chromosome karyotype of either spouse.

### Randomization and blinding

**Figure 1:**
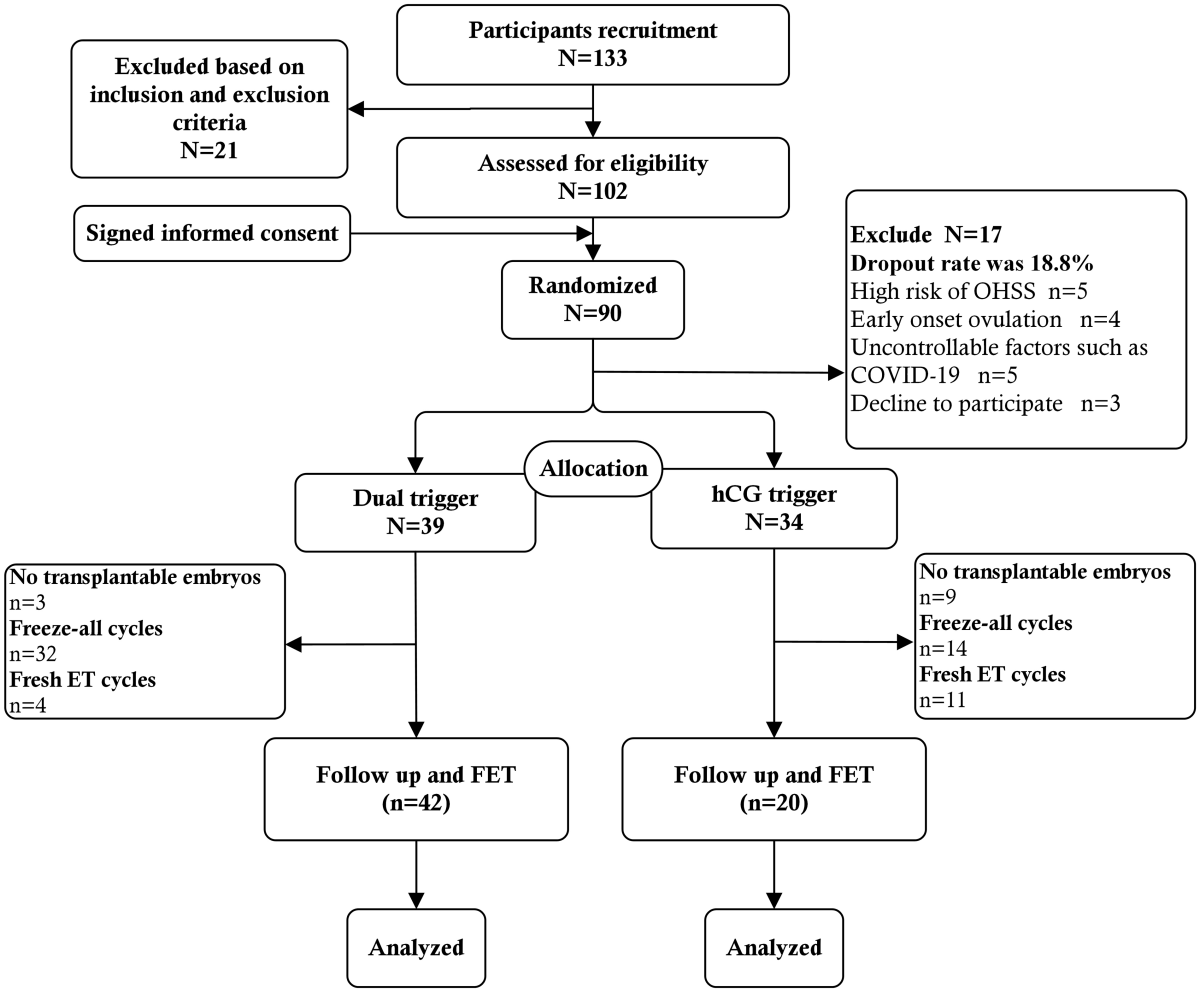
Flowchart of study population recruitment.

Randomization was commenced on the trigger day. A random sequence of codes was used to assign individuals to one of two groups, A or B, in a 1:1 ratio by computer. A central randomization database was established to store the randomization scheme.^1^ The online randomization procedure was operated by a data specialist who was not involved in patient recruitment and clinical management. Physicians were informed by e-mail of the allocation results after randomization. As a result, the operation ensured that allocation concealment was maintained since the service did not reveal the allocation until after the randomization process, i.e., after the baseline visit. Considering the nature of the intervention, we did not blind physicians and participants to the intervention. However, the trial outcome assessors were blinded to the assigned groups (Figure 1).

### Controlled ovarian stimulation protocol

Standard ovarian stimulation protocol with gonadotrophins was performed using a GnRH-ant protocol. Ovarian stimulation with 150–225 IU/day of recombinant FSH (Puregon, Merck Sharp & Dohme B.V., Haarlem, Netherlands) was started on the 3rd day of the menstrual cycle. The patients’ attending physicians determined the starting dose of Gn based on their age and BMI before participation in the study and dynamically monitor the ovarian response based on the results of serial transvaginal ultrasound follicle measurements and assays of serum E_2_, progesterone (P_4_), LH. The dose of Gn was adjusted according to the subject’s response. When ovarian stimulation reached the fifth day, GnRH-ant (0.25 mg, Cetrorelix, Merck Serono, Darmstadt, Germany) was added and continued until trigger day. When 2 follicles were ≥ 18 mm in diameter or when 3 follicles were ≥ 17 mm in diameter, triggering was performed. According to the results of randomization, patients were divided into the following two groups: (1) hCG-only trigger: Patients were triggered with recombinant hCG (r-hCG, 6,500 IU, Ovidrel, European Serono, France) 36 h before OPU; and (2) Dual trigger: Patients were triggered with co-administration of 0.2 mg GnRH-a (0.1 mg, Diphereline, France, Epson) and r-hCG (6,500 IU), 40 and 34 h prior to OPU, respectively. Oocyte retrieval was performed by transvaginal puncture under transvaginal ultrasound guidance.

### Embryo culture

Cumulus cells were enzymatically removed from oocytes and mature oocytes were subjected to intracytoplasmic sperm injection. In an environment of 5.0% O_2_ and 5.6% CO_2_, the pre-equilibrated embryo Petri dishes were used to culture zoosperm. According to Gardner’s criteria, embryo morphology and quality were evaluated. When selecting embryos for vitrification or transfer, it should focus on no fewer than six blastomeres with ≤20% fragmentation, which indicates top quality. Embryos with a fragmentation rate between 20 and 50% were not transferred or vitrified unless they reach the 8-cell stage on day 3. In our center, we have consistently adhered to the principle of transferring one top-quality day 3 embryo or two suboptimal embryos at the cleavage stage. Suboptimal embryos were defined as blastomeres of unequal size, irregular shape, and granular cytoplasm on the third day, with fragmentation rates between 20 and 50%. Fresh embryo transfer would be cancelled if met: Endometrial thickness less than 7 mm on trigger day; Serum P_4_ levels greater than 1.5 ng/mL on trigger day; High OHSS risk, i.e., retrieval of more than 15 oocytes and serum E_2_ concentrations higher than 4,000 pg./mL on trigger day, etc.

### Endometrial preparation protocol for FET

Transvaginal ultrasound and serum hormone measurements (LH, E_2_, and P_4_) were performed on days 8 through 10 of the menstrual cycle, depending on the duration of the menstrual cycle, to track endometrial thickness and follicle size until ovulation triggering conditions were reached. A single intramuscular injection of 4,000 IU hCG (Lizhu Pharmaceutical Trading Co., China) was used to trigger ovulation when the dominant follicle diameter was more than 17 mm, the endometrial thickness was more than 7 mm, P_4_ was less than 1.5 ng/mL, and E_2_ was more than 150 pg./mL. At approximately 9:00 am, the hCG injection was administered. Patients who had unanticipated spontaneous ovulation while being monitored, as well as those who had no prominent follicles by day 25 of the menstrual cycle, were excluded.

### Luteal phase support protocols

Routine luteal phase support was given after embryo transfer (ET), using intramuscular P_4_ (Zhejiang Xianju Pharmaceutical Co., Ltd.) injection 40 mg/day, P_4_ vaginal sustained release gel (8% Crinone, Moxerano) 90 mg/day, or oral P_4_ 10mg three times a day (dydrogesterone, Abbott Laboratories biologicals), or in combination, continued from the day of ET until 10 weeks of gestation. The manner of P_4_ administration is determined by the clinical preferences of both physicians and patients.

### Study endpoints and definitions

The primary outcome was oocytes maturation rate (i.e., the percentage of the number of MII oocytes over the total number of oocytes retrieved).

The secondary outcomes are number of oocytes retrieved, normal fertilization rate (the proportion of oocytes that become fertilized), number of two-pronuclear (2PN) embryos, number of D3 top quality embryos (TQE), number of D3 transferable embryos, number of remaining frozen embryos, cumulative clinical pregnancy rate and cumulative live birth rate (LBR). Top quality embryo was defined as seven or more blastomeres of uniform size and fragmentation rate less than 20% on day three. Clinical pregnancy was defined as the appearance of a gestational sac and fetal heartbeat detected by transvaginal ultrasonography. Cumulative clinical pregnancy rate and LBR were defined as the proportion of participants with clinical pregnancy or live birth after 1 year of follow-up.

### Data statistical analysis

This clinical study was a superiority, randomized parallel controlled trial. The primary outcome measurement was MII oocytes rate. According to previous data of our center, the mean estimated rate of MII oocytes in dual trigger group was approximately 70%, using GnRH-a (40 h before OPU) + hCG (34 h before OPU), and 30% in hCG trigger group, with standard deviations of 40% for each group. The sample size of 40 individuals for each group was calculated by PASS 15.0 (NCSS, LLC. Kaysville, Utah, United States) assuming *α* = 0.05 (two-sided) and *β* = 0.10 (90% power). Suppose that a dropout rate of 10%, we calculated that the sample size for each group was 45 individuals. Ultimately, the RCT need to recruit a total of 90 individuals.

Because the randomization was implemented on the trigger day, recruited participants who dropped out of trial during the ovarian stimulation process were not randomized. Therefore, only outcome variables were analyzed for subjects randomized to the study protocol (per protocol analysis). All the data were analyzed with SPSS version 26.0. Data are presented as mean ± standard deviation (Mean ± SD) for continuous variables and as frequency (percentage) [n (%)] for categorical data. According to the normality and variance of the data, the continuous data were analyzed by use of Student’s *t*-test or Mann–Whitney U-test. When data did not conform to normal distribution, a nonparametric test was used, which were expressed as the median (interquartile range) [M (IQR)]. Categorical data were analyzed using chi square and Fisher’s exact tests with an expected frequency of less than 5. *p* < 0.05 indicated that the difference was statistically significant.

## Results

### Baseline characteristics

A total of 133 patients were recruited at the time of presentation, and 21 were subsequently excluded based on the inclusion criteria and exclusion criteria. Twelve patients voluntarily abandoned participation in the study. Ultimately 90 patients signed informed consent to participate in the study at the start of stimulation. Among them, 5 patients who had high risk of OHSS (i.e., more than 20 follicles over 10 mm in diameter) in the ovarian stimulation process were excluded. Moreover, GnRH-a trigger and freeze-all strategy were used. One patient who had early follicular ovulation, and 8 patients who had broken off ovarian stimulation due to uncontrollable factors such as COVID-19 were also not included. The remaining 3 patients decline to participate. No cases of early onset ovulation were observed after trigger. The actual dropout rate was 18.8%. Finally, a total of 73 patients were included in the analysis. Thirty-four patients were assigned to the hCG trigger group and 39 patients were assigned to the dual trigger group. The baseline characteristics and demographics did not differ significantly between the dual trigger and hCG trigger groups in terms of age, BMI, AFC, basal sex hormone level, duration type of infertility and reason for infertility (Table 1). Although there were no significant differences in the total dose of Gn between the two groups, the total duration of ovarian stimulation was higher in dual trigger group (Table 1).

**Table 1:** Participants demographics characteristics of study participants.

	hCG trigger (*n* = 34)	Dual trigger (*n* = 39)	*p* value
Age (yrs.)	30.97 ± 3.65	31.26 ± 4.05	0.754^a^
Duration of infertility (yrs.)	3.00 (2.75)	3.00 (2.50)	0.537^b^
BMI (kg/m^2^)	22.1 (4.00)	22.5 (4.65)	0.847^b^
AMH (ng/ml)	3.58 (1.41)	2.96 (1.46)	0.174^b^
AFC (*n*)	12.5 (7.0)	13.0 (6.0)	0.233^b^
Basal FSH (IU/L)	6.96 (1.83)	6.46 (2.16)	0.071^b^
Basal LH (IU/L)	4.33 (1.98)	4.95 (3.65)	0.398^b^
Basal E_2_ (pg/ml)	38.2 (16.1)	40.0 (23.3)	0.283^b^
Basal P_4_ (ng/ml)	0.54 (0.29)	0.54 (0.42)	0.956^b^
Type of infertility (%)			0.094^c^
Primary	24/34 (70.6)	19/39 (48.7)	
Secondary	10/34 (29.4)	20/39 (51.3)	
Reason for infertility(%)			0.387^c^
Tubal infertility	18/34(52.9)	14/39 (35.9)	
Male factor	16/34(47.1)	24/39 (61.5)	
Unexplained infertility	0	1/39(2.6)	
Total duration of ovarian stimulation (d)	9 (1.75)	10 (2.00)	<0.001
Total dose of gonadotropin (IU)	1913 (543)	2025 (544)	0.110

BMI, body mass index; AMH, anti-Müllerian hormone; AFC, antral follicle count; FSH, follicle-stimulating hormone; LH, luteinizing hormone; E2, estradiol; P, progesterone. Data are presented as mean ± SD, the Median (IQR) or *n* (%).

a Student’s *t* test.

b Independent-Samples Mann–Whitney U-Test.

c Fisher’s exact test.

### Ovarian stimulation outcomes

Serum LH, E_2_ and P_4_ levels were not significantly different between the two groups on trigger day. The number of oocytes retrieved (15 [11.8] vs. 17 [12.5], *p* = 0.088) did not differ significantly between the two study groups, but there was a significant difference in number of MII oocytes retrieved (7.5 [4.0] vs. 15 [9.0], *p* < 0.001), MII oocytes rate (55.5% [19.8%] vs. 84.0% [14.0%], *p* < 0.001), normal fertilization rate (65.0% [39.5%] vs. 75.0% [33.5%], *p* = 0.032), number of 2PN embryos (3.0 [4.0] vs. 9.0 [7.5], *p* < 0.001), number of D3 TQE (0 [1.0] vs. 1 [3.0], *p* = 0.003), number of D3 transferable embryos (1.00 [3.75] vs. 4.0 [4.50], *p* < 0.001), and number of remaining frozen embryos (0 [2.0] vs. 4 [4.5], *p* < 0.001) between the hCG trigger and dual trigger groups (Table 2). Further, there was no difference in the incidence of no embryos available for transfer between patients with dual trigger and those with hCG trigger (26.5% vs. 7.7%, *p* = 0.055, see Table 2).

**Table 2:** Comparison of cycles characteristics between two study groups.

	hCG trigger (*n* = 34)	Dual trigger (*n* = 39)	*p* value*
LH on trigger day (IU/L)	2.05 (2.24)	1.68 (1.12)	0.248
E_2_ on trigger day (pg/ml)	3,061 (1385)	3,957 (2026)	0.146
P_4_ on trigger day (ng/ml)	1.23 (0.575)	1.07 (0.715)	0.286
Number of oocytes retrieved	15 (11.8)	17 (12.5)	0.088
Number of MII oocytes retrieved	7.50 (4.00)	15 (9.00)	<0.001
MII oocytes rate (%)	55.5 (19.8)	84.0 (14.0)	<0.001
Normal fertilization rate (%)	65.0 (39.5)	75.0 (33.5)	0.032
Number of 2PN embryos	3.0 (4.0)	9.0 (7.5)	<0.001
Number of D3 embryos available for transfer	1.0 (3.75)	4.0 (4.50)	<0.001
Number of D3 TQE	0 (1.00)	1.0 (3.00)	0.003
Number of remaining frozen embryos	0 (2.00)	4.0 (4.50)	<0.001
Cycles of no embryos available for transfer (%)	9/34 (26.5)	3/39 (7.7)	0.055

MII, metaphase II; 2PN, two-pronuclear; TQE, top quality embryos. Data are presented as the Median (interquartile range, IQR). * Independent-Samples Mann–Whitney U-Test.

### Pregnancy outcomes

Following fresh ET, there was no significant difference in the LBR (Table 3). Nevertheless, the cumulative pregnancy rate (10/25 [40.0%] versus 25/36 [69.4%], *p* = 0.035) and cumulative LBR (9/25 [36.0%] versus 24/36 [66.7%], *p* = 0.022) of dual trigger group was significantly higher than that of hCG trigger group. There was no significant difference between the two groups in the number of cycles which were used to calculate the cumulative pregnancy/live birth rate.

**Table 3:** Comparison of pregnancy outcomes between two study groups.

	hCG trigger (*n* = 34)	Dual trigger (*n* = 39)	*p* value*
Live birth rate per FreET (%)	4/11 (36.4)	2/4 (50.0)	1.000
Cumulative clinical pregnancy rate (%)	10/25 (40.0)	25/36 (69.4)	0.035
Cumulative live birth rate (%)	9/25 (36.0)	24/36 (66.7)	0.022
Cumulative number of cycles			0.612
1	21/25 (84.0)	27/35 (77.1)	
2	2/25 (8.0)	6/35 (17.1)	
≥3	2/25 (8.0)	2/35 (5.7)	

FreET, fresh embryo transfer. Data are *n* (%).* Fisher’s exact test.

## Discussion

This study shows that co-administration of GnRH-a and hCG for final oocyte maturation, 40 and 34 h prior to OPU, respectively (dual trigger), increased the number of MII oocytes retrieved and the rate of mature oocytes in patients with normal ovarian response who had a poor oocyte maturation rate (<50%) in the previous cycle.

Oocytes complete meiosis I and arrest at metaphase II until fertilization, at which point meiosis II is completed (15). Twenty-eight to thirty-eight hours after the onset of the LH surge preovulatory oocytes in metaphase II were obtained (16). After controlled ovarian stimulation, some of the oocytes retrieved are arrested at the germinal vesicle or metaphase I (MI) stage despite correct administration of hCG (17).The etiology of oocyte maturation arrest is complex. Recently, pathogenic variants in genes associated with oocyte maturation arrest have been identified. It might lead to meiotic arrest of oocytes by decreasing the amount of protein, disrupting microtubule formation, and impairing spindle assembly (18–20). Unfortunately, there is no effective treatment at present. When the percentage of meiotic competence failure oocytes was 25% or more, no pregnancy was achieved (21). A study (13) shows that when the oocyte maturation rate is low (<57.5%), it indicates an unfavorable IVF cycle outcome. As is well known, hCG has no FSH activity. Compared with hCG alone, GnRH-a trigger induces an increase in endogenous LH and FSH, which is similar to the surge of gonadotropin in the middle of the natural cycle. Fabris et al. demonstrated that in patients with high immature oocyte rate in the previous IVF cycle, the number of retrieved mature oocytes increases when the proportion of immature oocytes declines due to dual triggering (22). Our study reached the same conclusion, but differed in that the administration of GnRH-a and hCG did not occur simultaneously and prolonged the time between trigger and OPU. Dual trigger that can increase the number of mature oocytes and increase the rate of mature oocyte may be related to the surge of FSH. The surge in FSH stimulates the cumulus cells of oocyte-cumulus complexes to secrete a meiotic activating substance that allows the oocyte meiotic process to resume and activate cumulus expansion during the final stages of oocyte maturation (23, 24). Furthermore, FSH has been shown to promote the formation of LH receptor sites in granulosa cells and the development of the corpus luteum, which in turn promotes estrogen and P_4_ production (25).

Additionally, GnRH receptors have been found in a variety of human tissues, including the granulosa cells of the pre-ovulatory phase. In mammals, oocytes remain in the prophase of the first meiosis until the gonadotropin surge at puberty. The intra-oocyte concentration of cAMP and cGMP in this prolonged period prevents the resumption of meiosis in the oocyte. As a result of LH, cGMP levels decrease and meiosis resumes (26). It has been demonstrated that peripheral GnRH receptor activation decreases intracellular cAMP levels. Several genes are induced by GnRH that are involved in follicular rupture and oocyte maturation (27). The favorable results in our study could be attributed to the FSH surge and direct action of the agonist on the ovarian GnRH receptor. Perhaps, for unknown reasons, the LH pathway in the patient is blocked, and GnRH agonists activate a different pathway. Another possibility is that the interval between the ovulation trigger and oocyte retrieval is prolonged. In a natural cycle, the onset of LH surge occurs 34–36 h before follicular rupture. For optimal oocyte maturation, LH concentration must be maintained above a threshold for 14–27 h (28). Oocyte maturation and follicular rupture are time-dependent processes, which require different times in different patients. It is hypothesized that certain patients require a longer period of time for cumulus expansion, which allows the oocyte to detach from the follicular wall. In these cases, oocyte immaturity may result when aspiration occurs 36 h after hCG administration (29, 30).

Co-administration of GnRH-a and hCG as trigger was associated with increased embryo implantation (31). Endometrial receptivity may be improved by GnRH-a acting as an autocrine and (or) paracrine regulator (27). Cheon et al. suggested that an increased expression of endometrial GnRH-II peptide, noted during the early and mid-secretory phase, may play an important role in human embryo implantation (32). Co-administration of GnRH-a and hCG for final oocyte maturation not only compensated for luteal insufficiency after GnRH-a trigger but also improved patients cycle outcomes by improving endometrial receptivity. Consistent with the study findings that Decleer et al. (33) suggested that women who received dual triggering were more likely to have a surplus of frozen embryos. The studies by Griffin et al. and Gao et al. showed that the dual trigger did not improve patients cycle outcomes, which is in contrast to the findings of our study (17, 34). Sequential trigger is the co-administration of GnRH agonist and hCG for final oocyte maturation, 40 and 34 h prior to OPU, respectively. Double trigger is the simultaneous administration of GnRH agonist and hCG 36 h prior to OPU for final oocyte maturation. The dual trigger used in this RCT, namely the sequential trigger, differs from the double trigger in that it additionally prolongs the time between ovulation triggering and OPU. This later prolongation, may explain the beneficial effect in terms of both oocytes maturation and pregnancy rate.

In this RCT, it is undeniable that limitations remain, including the relatively small sample size and unexpected higher dropout rate. Although our included population was normal responders, the fact is that partial normal responders also face a high risk of OHSS in the course of ovarian stimulation. When this happened, they were excluded. Therefore, this study cannot discuss the relationship between dual trigger and OHSS incidence. Because too few patients underwent fresh ET, it was not powered to show a difference in the pregnancy outcomes with fresh ET. Moreover, we did not detect and analyze relevant gene mutations in this population due to cost–benefit considerations. Although the duration of Gn administration was increased by 1 day in the dual trigger group, the number of oocytes retrieved was not increased compared with that in the hCG trigger group. Moreover, no statistical difference was found in the dosage of Gn between the two groups. Therefore, this may not be the reason for the increase in the number of mature oocytes.

## Conclusion

In conclusion, we demonstrated that co-administration of GnRH-a and hCG for final oocyte maturation, 40 and 34 h prior to OPU, respectively, can increase MII oocyte rate and enable patients to obtain more embryos with higher quantity and quality. Moreover, the dual trigger was equally beneficial for pregnancy outcomes.

## Data availability statement

The original contributions presented in the study are included in the article/supplementary material, further inquiries can be directed to the corresponding authors.

## Ethics statement

The studies involving humans were approved by the reproductive ethics committee of the Affiliated Hospital of Shandong University of Traditional Chinese Medicine. The studies were conducted in accordance with the local legislation and institutional requirements. The participants provided their written informed consent to participate in this study.

## Author contributions

M-HY: Data curation, Formal analysis, Writing – original draft, Writing – review & editing. Z-GS: Funding acquisition, Supervision, Validation, Conceptualization, Writing – review & editing. J-YS: Conceptualization, Data curation, Writing – review & editing.

## Footnotes

^1^ www.medresman.org
